# The *Candida albicans* Inhibitory Activity of the Extract from Papaya (*Carica papaya* L.) Seed Relates to Mitochondria Dysfunction

**DOI:** 10.3390/ijms18091858

**Published:** 2017-08-25

**Authors:** Tao Zhang, Weijun Chen

**Affiliations:** 1 College of Food Science, Hainan University, Haikou 570228, China; tao_zhang0922@hotmail.com; 2 State Key Laboratory of Food Science and Technology, School of Food Science and Technology, Jiangnan University, Wuxi 214122, China

**Keywords:** papaya seed, *C. albicans*, benzyl isothiocyanate, mitochondria dysfunction, reactive oxygen species

## Abstract

The inhibitory activity of the papaya seed extract (PSE) on *Candida albicans* (*C. albicans*) was determined by turbidimetry method. The inhibitory mechanisms were also evaluated from the prospective of reactive oxygen species (ROS) generation, mitochondrial membrane potential (MMP) decrease, and the activities of four complex enzymes in mitochondria respiratory chain. Results obtained from this study indicated that the PSE exhibited an effective inhibitory activity on *C. albicans* and induced significant accumulation of ROS and collapse of MMP. The Complex I and Complex III exhibited continues significant decrease in mitochondrial enzyme activity assays, but the Complex II and Complex IV activities were not positively correlated. Furthermore, the GC-MS analysis demonstrated that the PSE represents a rich and high-purity source of benzyl isothiocyanate (BITC), which indicated the BITC may be responsible for the mitochondrial dysfunction.

## 1. Introduction

Papaya (*Carica papaya* L.) is an important economic fruit in both tropical and sub-tropical region [1]. Papaya fruits have amounts of carbohydrates and water, low in calories but rich in minerals and natural vitamins, particularly vitamin A and vitamin C [2]. The central cavity of the flesh contains large quantities of seeds that comprise about 15% of the wet weight of the papaya fruit [3]. The papaya seeds had a high content of lipid (29.16%) and protein (25.63%). The lipid in the papaya seeds is considered economically attractive for industrial extraction, especially when compared to conventional oilseed crops such as corn and soybean [4]. Traditionally, papaya seeds were used in parts of Asia and South America as a vermifugal [5] and seed preparations were also used in folk medicine due to its abortive properties to favor a good menstrual flow [6]. In recent years, many studies have been conducted to utilize the papaya seeds, and extensive research has been carried out on the use of the seeds as a source of oil [7,8,9], It was reported that the papaya seed oil had an interesting composition (72% of monounsaturated fatty acids with 71% oleic acid) and hence representing a promising source of oleic oil for various applications [10]. Furthermore, the papaya seed extract (PSE) has been pronounced with some special functions such as antibacterial [11], anti-fertility [12,13,14,15], antihelminthic and antiamoebic [16] activities. Previous studies have discovered many bioactive compounds existed in papaya seeds [17], including substantial amounts of carotenoid pigments, isothiocyanate and phenolic compounds [8]. However, the seeds of papaya were generally discarded and little attention was given for further application of these bioactive compounds.

*Candida albicans* (*C. albicans*), a common fungus, is frequently a benign member of the skin and mucosal flora. Research has shown that *C. albicans* could induce mucosal membranes diseases [18]. Antecedent colonization of mucosal surfaces with *C. albicans* could cause life-threatening infections in abnormal hosts. Approximately 75% of women will have one episode of candida vaginitis in their lifetime, with half having at least one recurrence [19], and invasive candidiasis is particularly common in intensive care units where mortality rates reach 45–49% [20]. Even worse, the resistance of pathogenic fungi, in particular *C. albicans* and non-*C. albicans* species isolated from patients, against antifungal agents has increased [21]. Based on the increasing side-effects of polyenes and azoles, novel antifungal therapies with fewer side effects on humans are urgently required for effective management of candidiasis [22].

Mitochondria dysfunction involves in many antifungal activities. In the endosymbiotic association of oxidative bacteria and most eukaryotic cells, mitochondria have become an increasingly important target for cellular research. Recently, the extract of papaya seeds was reported to exhibit an antifungal activity against *Candida albicans* [23,24]. However, possible mechanism of the inhibitory activity was ignored. The aim of this study was to highlight the potential compounds in the PSE as a new natural source for *C. albicans* inhibition. The inhibitory mechanisms were also evaluated from the prospective of reactive oxygen species (ROS) generation, mitochondrial membrane potential (MMP) decrease, and the activities of four complex enzymes in mitochondria.

## 2. Results

### 2.1. C. albicans Inhibitory

**Figure 1:**
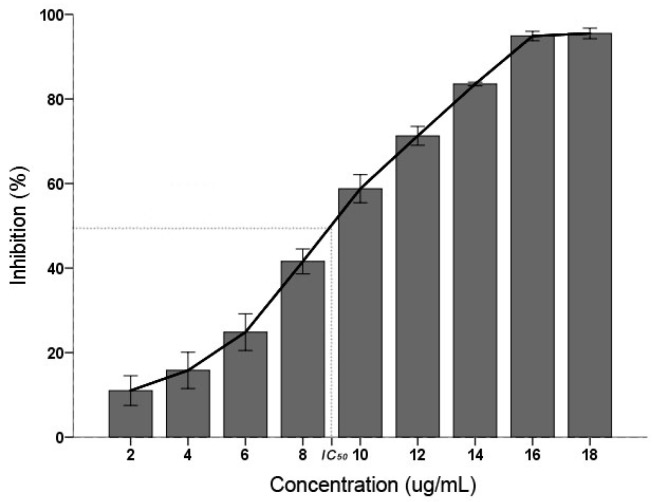
Effects of the inhibitory activity on the inhibition of *C. albicans*. Inhibition activity was determined after 24 h of treatment with concentrations of 2–18 µg/mL. Data (*n* = 3) are expressed as mean ± SD. The statistical significant of differences was calculated using one-way ANOVA with Bonferroni’s post hoc test.

*C. albicans* inhibitory result displayed that the PSE have an effective inhibitory activity (Figure 1). The inhibition activity were increased with the increasing concentration (*p* < 0.01): when the concentration was 18 µg/mL, the inhibition was 95.08 ± 0.62% and found not significantly different (*p* > 0.05) with 16 µg/mL. According to the Clinical and Laboratory Standards Institute (CLSI) interpretative guidelines on antifungal susceptibility testing, the half maximal inhibitory concentration (IC_50_) between 16 and 32 µg/mL is considered as susceptible dose dependent [25]. In this study, the IC_50_ of PSE was calculated as 9.36 µg/mL. It was suggested that the PSE exhibited a remarkable inhibitory activity on *C. albicans*.

### 2.2. ROS Increase

**Figure 2:**
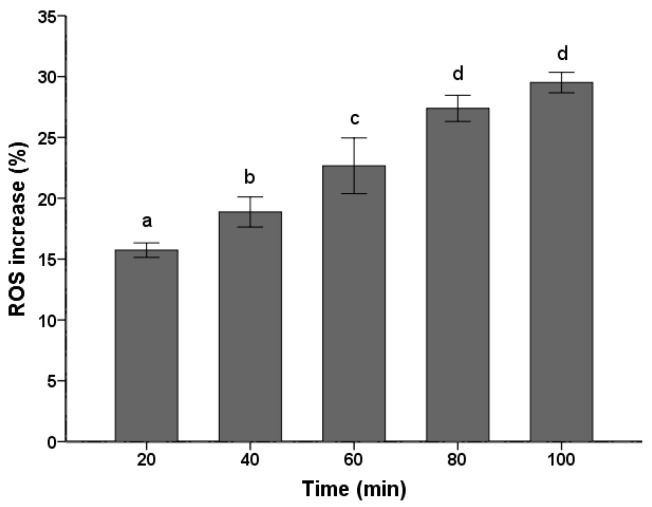
Effects of the PSE on the increase of ROS in *C. albicans* cells. Cells were treated with 20 µg/mL extract for 20 to 100 min, stained with DCFH-DA (10 µM/L) for 15 min and analyzed by fluorescence. Fluorescence intensity is an indication of ROS levels in *C. albicans* cells. Data (*n* = 3) are expressed as mean ± SD. The statistical significant of differences was calculated using one-way ANOVA with Bonferroni’s post hoc test. Different letters represent significant difference (*p* < 0.05).

ROS production and accumulation is a common denominator in many diseases. Environmental insults can lead to severe cellular damage leading to physiological dysfunction and cell death in virtually all aerobes. The fluorescent probe DCFH-DA has been widely used for measurement of ROS formation in cells [26,27]. As shown in Figure 2, the PSE caused a continuous increase in ROS level of *C. albicans* cells. With the treatment time prolonged, there was significant (*p* < 0.05) increase in ROS accumulation. The ROS increased to the maximum level (29.51 ± 0.84%) when treated for 100 min, which was corresponded to 1.30 times compared with the control treated for 0 min. Previous studies have shown that *C. albicans* treated with 8 µg/mL plagiochin E, ROS would increased 1.28 times after two hours [26]. Though concentration in this study changed, the result still reflected that the PSE exhibited a strong vitality for ROS accumulation in *C. albicans*. By taking the ROS generation in previous study as a reference [26], the ROS increase in levels (Figure 2) were not low, especially when treated for 20 min.

### 2.3. MMP Decrease

**Figure 3:**
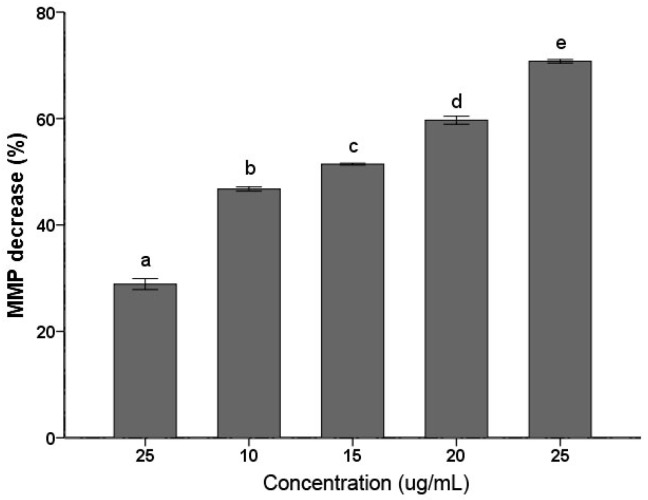
Effects of the PSE on the loss of mitochondrial membrane potential (MMP) in *C. albicans* cells. Cells were treated with or without the extract for 24 h. After incubation, cells were stained with Rhodamine 123 (20 µM/L) for 30 min at 37 °C and analyzed by fluorescence. The reduced fluorescence of Rhodamine 12 was determined as the reduced MMP. Data (*n* = 3) are expressed as mean ± SD. The statistical significant of differences was calculated using one-way ANOVA with Bonferroni’s post hoc test. Different letters represent significant difference (*p* < 0.05).

Decrease of MMP has been considered a characteristic feature in the early stage during apoptosis. As shown in Figure 3, *C. albicans* cells treated with the PSE exhibited significant (*p* < 0.01) decrease in MMP at 5–25 µg/mL. The decrease in MMP rates was increased with the increasing of the concentration. The 25 µg/mL treatment group rapidly increased the proportion of Rhodamine 123 negative cell to 79.27 ± 1.10% (*p* < 0.01). The data indicated that *C. albicans* cell exposure to the extract resulted in MMP collapse. MMP depolarization promotes ROS production. The results obtained were consistent with previous results of similar studies about medioresinol leading to MMP decrease of *C. albicans* [26].

### 2.4. The Activity of Mitochondrial Complex Enzymes

**Figure 4:**
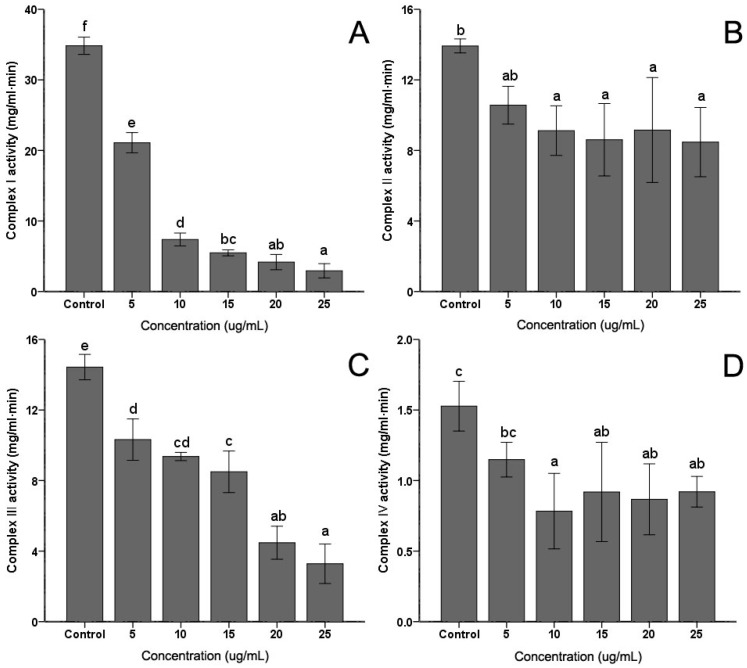
Effects of the PSE on the activities of mitochondrial complex enzymes in *C. albicans* cells. *C. albicans* cells were pretreated with different concentrations (0 to 25 µg/mL, 24 h): (**A**) Complex I; (**B**) Complex II; (**C**) Complex III; and (**D**) Complex IV. Data (*n* = 3) are expressed as mean ± SD. The statistical significant of differences was calculated using one-way ANOVA with Bonferroni’s post hoc test. Different letters represent significant difference (*p* < 0.05).

The activities of mitochondrial complex enzymes are the most commonly assayed parameters of mitochondrial functions. Activities of the four complexes in the electron transfer chain in *C. albicans* were measured. As shown in Figure 4, the extract induced some decrease in Complex I, Complex II, Complex III and Complex IV activities to different degrees. However, with the increasing of the PSE concentration, only the activities of Complex I (Figure 4A) and Complex III (Figure 4C) exhibited continued significant decreasing (*p* < 0.05). No obvious and sustaining changes (*p* > 0.05) were found in Complex II (Figure 4B) or Complex IV (Figure 4D) when concentration was higher than 10 µg/mL. With 25 µg/mL PSE treatment, the activities of four complex enzymes reached the lowest activities, 9.47%, 68.10%, 25.50% and 67.32%, respectively (*p* < 0.01).

### 2.5. Chemical Composition of the Extract

**Figure 5:**
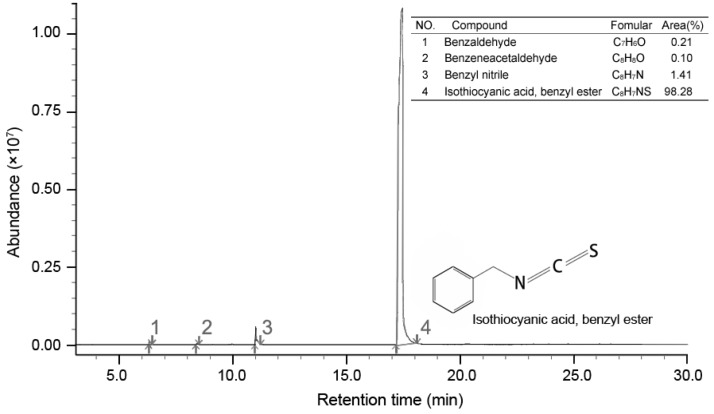
Total ion-current chromatogram of PSE by GC-MS.

GC-MS analysis result showed four compounds were identified as constituents of PSE, as presented in Figure 5. It was shown that PSE represented a rich and high-purity source of benzyl isothiocyanate (BITC, 98.28%). Other compounds, such as benzeneacetaldehyde (0.10%), benzyl nitrile (1.41%) and benzaldeehyde (0.21%), were also found. Previous studies on the composition of papaya seed extract demonstrated that BITC was the major bioactive compound [28]. The content of BITC could reach 99.36% [23]. Several methodologies have been reproted to obtain a high content of BITC [28,29,30]. BITC is a non-polar substance and mainly extracted by organic solvent in the mentioned previous sdudies. In contrast to solvent extract, steam distillation-extraction could get rid of the less volatile material and the purification process become easier. BITC are thought to be the bioactive compounds in PSE, responsible for the anthelmintic effect. However, the manner in which BITC is handled by the body is unclear. It seems that more studies need to be done to characterize the side effects of BITC before it can be firmly concluded that it is safe for further use.

## 3. Discussion

Many kinds of plants have been widely used for the treatment of various diseases for a very long time. Recently, interest in drugs of plant origin has significantly increased due to the less harmful side effects and wider availability. Research is being conducted all over the world to determine whether plants that are traditionally used in the treatment of various diseases are appropriate for intended use [31]. In this study, the data indicated that PSE could be utilized as an effective inhibitory medicine of *C. albicans* (Figure 1) owing to its high content of BITC (Figure 5). Previous studies have shown BITC is the major principle in papaya seeds [17]. This study obtained a high purity of BITC which was in good accordance with previous studies [29]. Furthermore, BITC is a volatile and relatively insoluble in water, the advantages of steam distillation extraction method is that BITC and volatile oil would be extracted while non-volatile oil and other impurities would be excluded. Thus, distillation of this compound from an aqueous solution with high purity in the current condition was not surprising. This study provided a preparation method of BITC for intensive research or industrial production. With azoles resistance *C. albicans* strains emerging in immune-compromised patients [32], the PSE may also serve as a novel drug and give a promising prospect for cardiac diseases treatment. However, intensive studies need to be done to investigate the side effects of BITC before firmly concluding that BITC is safe for further use.

Oxidative stress was considered e an important condition to promote cell death in response to a variety of signals and psychophysiological situations. ROS, which is predominantly produced in the mitochondria, if excessive, may lead to the free radical attack of membrane phospholipids and loss of mitochondrial membrane potential. Consequently, releases apoptosis-inducing factors that activate caspase cascades and cause nuclear condensation [33]. In this study, it was illustrated that the extract induced rapid and significant ROS generation in *C. albicans* cells (Figure 2). The results corroborate the similar conclusion obtained before [26], which reported that plagiochin E induced ROS accumulation in *C. albicans*. In addition, this study confirmed that ROS formation was the major event in BITC induced killing of *C. albicans*. Meanwhile, researches demonstrated that the antifungal action of many antifungal agents such as miconazole [34], indole-3-carbinol [35], and plagiochin E [26] are involved in the induction of ROS formation in *C. albicans*. Thus, the results were supporting the hypothesis that the rapid generation of ROS plays an important role in *C. albicans* cell death*.* Moreover, ROS generation is also correlated with the decrease of MMP. A rapid collapse of MMP was always found in some anticancer compounds induced apoptosis in cells [36]. The data in this study clearly show that the treatment with the PSE could lead to a loss of MMP (Figure 3). In many systems, cell death was associated with the loss of MMP, which was regarded as a limiting factor in that process. Reduction of the MMP is among the changes encountered during early reversible stages of deaths and is preceded by cytochrome c release in several cell types [37]. The result was in accordance with previous studies which suggested that a breakdown of MMP related to *C. albicans* cell deaths [26,35].

To investigate the mechanism of ROS accumulation and MMP decrease induced by PSE in *C. albicans* cells, the activities of four mitochondria complex enzymes in *C. albicans* cells (Figure 4) were evaluated. It was found that the Complex I and Complex III activities were significantly decreased, but Complex II and Complex IV activities were not positively correlated with the concentration. The changing of the enzyme activities indicated that PSE induced *C. albicans* death relates to the Complex I and Complex III dysfunctions. Mitochondria are an essential part of normal cellular function, particularly in the process of oxidizing carbon substrates to generate intracellular energy exchange. Changes of the mitochondrial protein activity were crucial in a number of cellular processes including the permeability transition and cell death. The influence of substances on mitochondria respiratory complex caused by drugs has already been reported [38]. The results were in accordance with previous findings: a reduction in the activities of Complex I and Complex III induced by ROS production could lead to an oxidation of these proteins [39]. On the other hand, an increase in activity of these enzymes may also correspond to an increase in oxidative stress. Furthermore, studies showed that BITC targeted mitochondria respiratory chain to trigger ROS-dependent apoptosis [40]. Generation of ROS by BITC led to induction of apoptosis in cancer cells [41,42]. These finds also proved the role of BITC in ROS production and mitochondrial dysfunction in cells.

**Figure 6:**
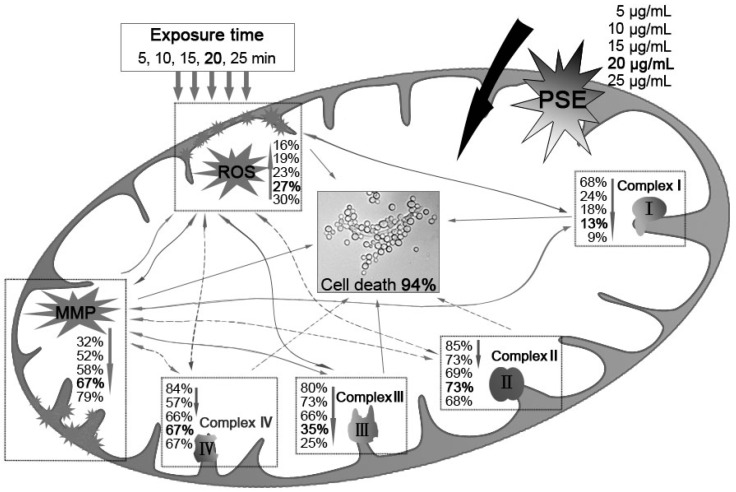
The *C. albicans* inhibitory mechanism of BITC. BITC inhibition correlated exponentially with MMP decay, ROS accumulation and the decreasing activities of mitochondria complex enzymes. Bold value was defined as the data when a significant cell death (94%) was induced. Real lines with arrows represent a significant and direct correlation, and dotted lines mean an uncertain relationship that requires further study. The percentages beside express the decrease or increase rates caused by treatment of different concentrations of PSE compared to the control.

Previous studies suggested that membrane lipid peroxidation and subsequent membrane dysfunction observed in cyanide intoxication is related in part to a compromised antioxidant defense. Given that cyanic acid group compounds are well known for their activities against mitochondrial electron transport chain Complex II and Complex IV, cyanide could increase the generation of ROS in mitochondria. Furthermore, it has been considered that enzyme activity of the mitochondria protein was inhibited by potassium cyanide. PSE may interfere with anti-ROS enzyme. Thus, the *C. albicans* inhibitory mechanism of the PSE can be illustrated as in Figure 6. First, BITC, the major compound in the PSE, exerted its antifungal activity through ROS accumulation and MMP depolarization in *C. albicans*. Then, the activation of mitochondrial apoptosis pathway, including Complex I and Complex III dysfunctions, was initiated. However, the detailed relationship among Complex I, Complex III and ROS accumulation or MMP decreasing is still under study. Further investigation is needed to elaborate why Complex II and Complex IV were not distinctly affected. In general, the results indicated the PSE could serve as a novel drug for cardiac diseases.

## 4. Materials and Methods

### 4.1. Plant Material and Extraction

The papaya seeds were purchased from local market and identified at Tropical Oil Crops Research Institute, Ministry of Agriculture, Wenchang China. After crushed, 100 g papaya seeds were placed in a round bottomed flask then steam distillation at 100 °C allowed for two hours. After centrifugation for 10 min at 5000× *g*, anhydrous sodium sulfate was added to remove water and then stored at 4 °C in the dark. The extract yield was found to be 0.14 ± 0.02% (*w*/*w*) as calculated on dry weight with a density of 1.12 g/mL.

### 4.2. Chemical and Reagents

Sodium succinate, rotenone, cytochrome c, coenzyme Q_1_, coenzyme Q_10_, antimycin A, decylubiquinol, 2′,7′-dichlorofluorescin diacetate (DCFH-DA) and Rhodamine 123 were purchased from Sigma (St. Louis, MO, USA). Tris base and NADH were purchased from Amersco Inc. (Palm Harbor, FL, USA). 2,6-Dichlorophenolindophenol (DCPIP) was purchased from Merck & Co. Inc. (Kenilworth, NJ, USA). Bovine serum albumin (BSA) for protein quantification was purchased from Sinopharm Chemical Reagent Co., Ltd. (Shanghai, China). All the other reagents used were of reagent grade and prepared in double-distilled water.

### 4.3. Microorganism and Culture Condition

The strain *C. albicans* was obtained from the American Type Culture Collection (ATCC). Cultures were maintained on a Sabouraud’s glucose medium (10 g/L of peptone, 20 g/L of agar, 40 g/L of glucose). The culture temperature was maintained at 32 °C and conducted at free pH with 300 rpm.

### 4.4. C. albicans Inhibitory Activity

*C. albicans* inhibitory activity was determined by a turbidimetry assay [43]. The extract solution (3.6 mg PSE dissolved in 1 mL ethanol and diluted with water to 100 mL) was mixed with a 12-hour-old culture of *C. albicans* (CFU = 10^6^) on yeast extract peptone dextrose (YPD) broth (20 g/L of peptone, 10 g/L of yeast extract, 20 g/L of glucose) and the final PSE concentration varying from 2 to 18 μg/mL were added to 96-wells plate. After the microplates were incubated at 30 °C for 24 h, the absorbance at 600 nm was measured. The inhibitory activity was calculated with the following equation:

*Inhibition* (%) = (*A*_1_ − *A*)/(*A*_1_ − *A*_0_) × 100

where *A* is the absorbance of the sample with the treatment of PSE; *A*_1_ is the absorbance without PSE treatment; and *A*_0_ is the absorbance of the culture medium.

### 4.5. Isolation of Mitochondria

Isolation of mitochondria were performed according to the method of Niimi with slight modifications [44]. Cells grown in YPD broth at 30 °C to early stationary phase were cultured, diluted to 5 × 10^6^ CFU/mL with fresh YPD broth at 30 °C for 24 h, after centrifugation at 5000× *g* for 10 min, the pellet was resuspened in homogenization medium (50 mM Tris-HCl, 2 mM EDTA, pH 7.5) and homogenized. Then was supplemented with 2% glucose before freezing and thawing three times repeatedly and centrifuged at 2000× *g* for 10 min to remove the cell debris and unbroken cells. The supernatants were collected and centrifuged at 12,000× *g* for 45 min. Then the pellet was resuspened in buffer (50 mM Tris-HCl, 2 mM EDTA, pH 5.0) incubated for 5 min, and centrifuged at 5000× *g* for 30 s. The pellet was collected as mitochondria resuspened in 50 mM Tris-HCl (pH 7.5)–2 mM EDTA-20% (*v*/*v*) glycerol then stored at −30 °C. Finally, the protein level was determined by Coomassie Brilliant Blue method.

### 4.6. Measurement of Reactive Oxygen Species (ROS) Formation

Generation of ROS was measured by the oxidative-sensitive fluorescent probe DCFH-DA [45]. Intracellular ROS can oxidize DCFH-DA to the highly fluorescent compound dichlorofluorescein. At 20, 40, 60, 80 and 100 min following the treatment with 20 μg/mL PSE, the medium was aspirated and replaced by DCFH-DA (10 μM) for a further 30 min at 37 °C. The cells were collected and then analyzed by fluorescence microplate reader.

### 4.7. Measurement of Mitochondrial Membrane Potential (MMP)

Depolarization of MMP during cell apoptosis results in the loss of Rhodamine 123 from the mitochondria and a decrease in intracellular fluorescence intensity. The effects of the PSE on the MMP was measured by fluorescence microscopy using Rhodamine 123 according to the procedure as described previously [36]. Briefly, at 24 h after the treatment with 0–25 μg/mL PSE, the cells were harvested and washed twice with Tris-HCl (50 mM, pH 7.5), incubated with 10 μM Rhodamine 123 for 30 min at 30 °C in the dark and gently washed three times with Tris-HCl. The intracellular fluorescence intensity was analyzed.

### 4.8. Assay for Mitochondrial Enzyme Activities

Before analysis, the mitochondrial samples were freeze-thawed and gently shaken three times to ensure mitochondrial reached the maximum activity. The NADH-ubiquinone reductase (Complex I) activity was determined by monitoring the reduction of DCPIP at 600 nm upon addition of assay buffer (10× buffer containing 0.5 M Tris-HCl, 2 mM NaN_3_, 10 μM antimycin A, 1% BSA, 0.5 mM coenzyme Q_1_, pH 8.1). The reaction was started by adding 200 μM NADH and scanned for 2 min. Rotenone (3 μM) was added to the reaction system as blank control [46]. Succinate-ubiquinone reductase (Complex II) was assayed in the assay buffer (10× buffer containing 0.5 M phosphate buffer, 1% BSA, 10 μM antimycin A, 2 mM NaN_3_, 0.5 mM coenzyme Q_1_, pH 7.8). The reaction was started with 10 mM succinate performed as described [47]. Ubiquinol-cytochrome c reductase (Complex III) was assayed by monitoring the reduction rate of cytochrome c at 550 nm upon the addition of assay buffer (0.5 M Tris-HCl, 2 mM NaN_3_, 0.8% Tween-20, 1% BSA, 2 mM decylubiquinol, pH 7.8) with 40 μM cytochrome c [38]. Cytochrome c oxidase (Complex IV) was assayed by the rate of oxygen consumption. Assay buffer contained 50 mM phosphate buffer, pH 7.0, 0.1% BSA, 0.2% Tween-20 and 30 µg/mL of mitochondrial protein. The non-enzymic rate of oxygen consumption was recorded before starting the reaction by addition of 40 μM reduced cytochrome c and scanned at 550 nm for 2 min [48]. All assays were conducted at 30 °C.

### 4.9. GC-MS Analysis

The components of the PSE were identified by GC-MS analysis as described previously [17]. The GC-MS was carried on a Varian Gas Chromatograph series 3800 fitted with a DB-5 capillary column (30 m × 0.32 mm, film thickness 0.25 μm) coupled with a 4000 series mass detector under the following conditions: injection volume 0.5 μL with split ratio 1:60, helium as carrier gas at 1.0 mL/min constant flow mode, injector temperature 250 °C, oven temperature was programmed from 50 to 280 °C at 5 °C/min. Mass spectra: electron impact (EI+) mode, 70 eV and ion source temperature 250 °C. Mass spectra were recorded over 40–600 a.m.u range.

### 4.10. Statistical Analysis

Each experiment was performed in triplicate, and statistical comparisons of the results were calculated using one-way ANOVA. Analysis of variance for individual parameters was performed based on mean values to determine the significance at *p* *<* 0.05. Data were expressed as means ± standard deviations.

## 5. Conclusions

In this study, the results have demonstrated that the extract from papaya could induce apoptosis in *C. albicans* cells significantly. In the apoptotic process, the extract induced intracellular accumulation of ROS followed by the collapse of MMP, then further leaded to the decreasing of the mitochondria Complex I and Complex III activities. Collectively, the results indicated a role of the extract as a novel drug for cardiac diseases which is a rich and high-purity source of BITC.
